# High-quality genome sequence of five Antarctic actinomycetes: Micromonospora ureilytica DSM 120150, Nocardiopsis akebiae DSM 120151, Streptomyces fildesensis DSM 41987T, Streptomyces hypolithicus DSM 41950T and Streptomyces albidoflavus DSM 120149

**DOI:** 10.1099/acmi.0.001141.v3

**Published:** 2026-02-12

**Authors:** Ulrike Tarazona Janampa, Meike Döppner, Jolantha Swiderski, Imen Nouioui, Cathrin Spröer, Boyke Bunk, Yvonne Mast

**Affiliations:** 1 Leibniz Institute DSMZ - German Collection of Microorganisms and Cell Cultures, Inhoffenstraße 7B, 38124 Braunschweig, Germany; 2 Universidad Científica del Sur, Antigua Panamericana Sur Km 19, 15067 Lima, Peru; 3 Braunschweig Integrated Centre of Systems Biology (BRICS), Rebenring 56, 38106 Braunschweig, Germany; 4 Technische Universität Braunschweig, Institut für Mikrobiologie, Rebenring 56, 38106 Braunschweig, Germany

**Keywords:** actinomycetes, antibiotics, Antarctica, genome sequence, natural products

## Abstract

*Micromonospora ureilytica* DSM 120150, *Nocardiopsis akebiae* DSM 120151, *Streptomyces fildesensis* DSM 41987^T^, *Streptomyces hypolithicus* DSM 41950^T^ and *Streptomyces albidoflavus* DSM 120149 are five Antarctic strains. Here, we present the high-quality genome sequences of DSM 120150, DSM 120151, DSM 41987^T^, DSM 41950^T^ and DSM 120149 with sizes of 7.51 Mbp, 6.90 Mbp, 8.91 Mbp, 6.01 Mbp and 6.85 Mbp, respectively.

## Data Summary

Genome sequence-related data availability is listed in Table 1 .

**Table 1.** Genome sequence, completeness and annotation data of Antarctic actinomycetes strains *M. ureilytica* DSM 120150, *N. akebiae* DSM 120151, *S. fildesensis* DSM 41987^T^, *S. hypolithicus* DSM 41950^T^ and *S. albidoflavus* DSM 120149

Attribute	Strain				
	DSM 120150	DSM 120151	DSM 41987**^T^**	DSM 41950**^T^**	DSM 120149
Genome length (bp)	7,508,249	6,900,836	8,913,051	6,014,151	6,852,435
Contigs	1	1	1	3	3
Genome coverage	76 ×	36 ×	101 ×	88 ×	121 ×
Average G+C content (%)	71	72.5	70.5	70	73.5
CDS	6,871	5,911	8,013	5,763	6,032
tRNAs	50	58	70	70	67
rRNAs	3, 3, 3 (5S, 16S, 23S)	5, 5, 5 (5S, 16S, 23S)	6, 6, 6 (5S, 16S, 23S)	7, 7, 7 (5S, 16S, 23S)	7, 7, 7 (5S, 16S, 23S)
RNA numbers	62	76	91	94	91
N50 (Mb)	7.5	6.9	8.9	6	6.9
CheckM completeness (%)	97.86	99.01	100	99.28	99.43
CheckM contamination (%)	3.54	3.97	1.26	0	0.38
Accession number	CP196338	CP196194	CP189853	CM128811	CP196343
BioProject number	PRJNA1293185	PRJNA1293186	PRJNA224116	PRJNA1334162	PRJNA1293190

## Announcement

The Antarctic habitat is one of the most unique and extreme environments on Earth [1]. Its harsh conditions, characterized by geographical and oceanographic isolation, freezing temperatures, long periods of darkness and limited accessibility, have created a habitat largely untouched by human influence [2,4]. The geographical and environmental isolation of Antarctica has allowed the development of highly specialized ecosystems, particularly in the benthic zones of the Southern Ocean, where life has adapted to thrive under some of the most challenging conditions on Earth [5]. Actinomycetes are one of the major sources of novel natural compounds [6]. Recent efforts in bioprospecting have focused on unique or extreme environments to increase the probability of finding structurally novel secondary metabolites [7,10]. As part of a bioprospecting effort targeting polar environments, five actinomycete strains were isolated from Antarctic quartz rock (DSM 41950^T^), soil (DSM 41987^T^) and marine sediment samples (DSM 120149, DSM 120150 and DSM 120151), and their genomes were sequenced.

Despite increasing interest in Antarctic micro-organisms, genome sequences of taxonomically well-characterized Antarctic actinomycetes, particularly type strains, remain limited. The five strains analysed here represent phylogenetically diverse actinomycetes isolated from distinct Antarctic habitats, including terrestrial, hypolithic and marine environments. The generation of high-quality genome sequences for these strains provides a valuable genomic resource that strengthens genome-based taxonomy and enables comparative analyses of biosynthetic gene cluster diversity across different actinomycete lineages.

Here, we present the annotated whole-genome sequences of five Antarctic strains, *Micromonospora ureilytica* DSM 120150, *Nocardiopsis akebiae* DSM 120151, *Streptomyces fildesensis* DSM 41987^T^, *Streptomyces hypolithicus* DSM 41950^T^ and *Streptomyces albidoflavus* DSM 120149, and describe their biosynthetic gene cluster content based on genome mining.

Strain DSM 120150 was cultivated on NL410 agar, strains DSM 120151 and DSM 120149 in R5 and S media and strains DSM 41950^T^ and DSM 41987^T^ in S-medium [11,12], with all cultures grown in 10 ml medium at 28 °C with shaking at 180 r.p.m. After 3 days, biomass was harvested for subsequent DNA extraction. Genomic DNA extraction was carried out using the MasterPure Gram-positive DNA purification kit (Lucigen, Middleton, WI, USA) according to the manufacturer’s instructions. DNA integrity was checked on a FemtoPulse system (Agilent, Santa Clara, CA, USA). The genomes of the strains were sequenced using the PacBio Sequel-*IIe* platform (Pacific Biosciences, Menlo Park, CA, USA). SMRTbell template libraries were prepared according to the instructions from Pacific Biosciences outlined in the Procedure and Checklist – Preparing Multiplexed Microbial Libraries Using SMRTbell Express Template Prep Kit v.2.0 [13]. Briefly, for preparation of 10 kb libraries, 1 µg genomic DNA was sheared using the Megaruptor v.3 (Diagenode, Denville, NJ, USA) according to the manufacturer’s instructions. The DNA was end-repaired and ligated to barcoded adapters, applying components from the SMRTbell Express Template Prep Kit 2.0 (Pacific Biosciences). Samples were pooled according to the calculations provided by the Microbial Multiplexing Calculator (Pacific Biosciences). Conditions for primer annealing and polymerase binding to purified SMRTbell templates were assessed with the Calculator in SMRT Link (Pacific Biosciences). Libraries were sequenced using one 15 h movie per SMRT cell. SAMtools v.1.12 was used to obtain PacBio reads in FASTQ format [14]. The quality of the PacBio reads was confirmed using LongQC v.1.2.0c [15], and no error correction was performed. PacBio reads were *de novo* assembled using Flye v.2.8.1 (https://github.com/fenderglass/Flye) with the command ‘flye--pacbio-raw’, which included circularization of the contigs. The presence of plasmids was analysed using RFPlasmid v0.0.18 [16], and assembly quality was evaluated using QUAST v4.4 [17]. The genome sequences of *M. ureilytica* DSM 120150, *N. akebiae* DSM 120151, *S. fildesensis* DSM 41987^T^, *S. hypolithicus* DSM 41950^T^ and *S. albidoflavus* DSM 120149 were deposited in GenBank under accession numbers CP196338 (DB10_83), CP196194 (M1B1), CP189853, CM128811 and CP196343 (DB7_152), respectively. Genome annotation was carried out with the NCBI Prokaryotic Genome Annotation Pipeline [18] (Table 1). Genome completeness and contamination were assessed with CheckM v1.0.18 [19]. All strains showed genome completeness above 97.86% and contamination below 3.97% (Table 1).

The phylogenetic relationship of the strains was analysed with the EzBioCloud server (https://www.ezbiocloud.net) [20], based on 16S rRNA gene sequences extracted from the genomes. The authenticity of the strains was confirmed by comparing genome-derived 16S rRNA gene sequences with those obtained by PCR. 16S phylogenetic analysis revealed that strains DSM 120150, DSM 120151, DSM 41987^T^, DSM 41950^T^ and DSM 120149 were most similar to *Micromonospora arida* LB32^T^ (MG725912) with 99.8% similarity [21], *Nocardiopsis dassonvillei* subsp. *crassaminis* D1^T^ (LR606207) with 99.91% [22], *S. fildesensis* GW25-5^T^ (DQ408297) with 99.72% [23], *S. hypolithicus* HSM10^T^ (EU196762) with 99.87% [24] and *Streptomyces pyxinicus* LP11^T^ (OL765279) with 98.89% similarity [25], respectively.

**Fig. 1.**
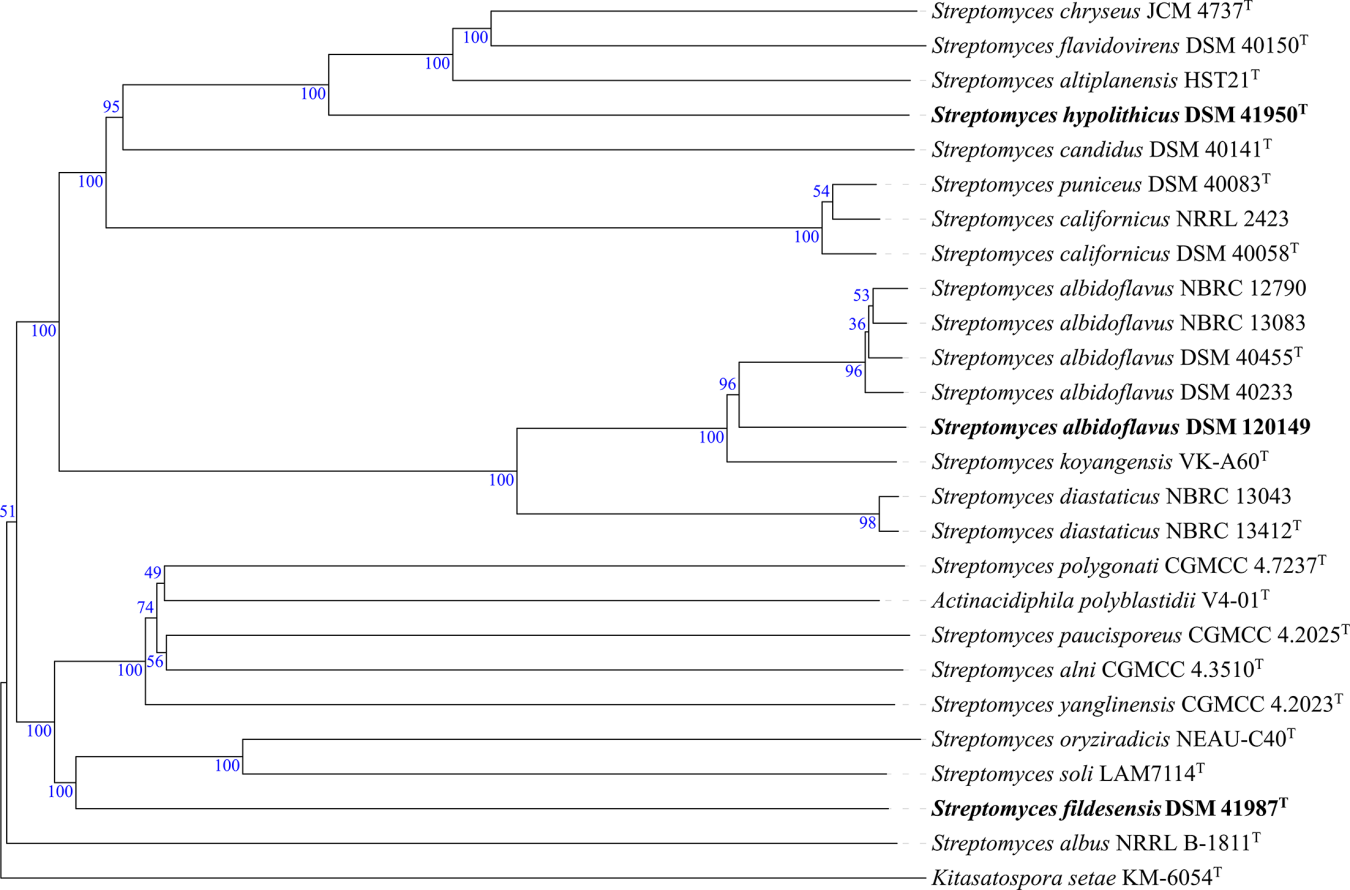
Phylogenetic tree of *S. fildesensis* DSM 41987ᵀ, *S. hypolithicus* DSM 41950ᵀ, and *S. albidoflavus* DSM 120149 inferred with FastME 2.1.6.1 [41] from Genome BLAST Distance Phylogeny (GBDP) distances calculated from genome sequences. The branch lengths are scaled in terms of the GBDP distance formula *d_5_*. The tree was rooted at the midpoint [42] and visually edited using Interactive Tree of Life (iTOL) v.7.2.1 [43].

**Fig. 2.**
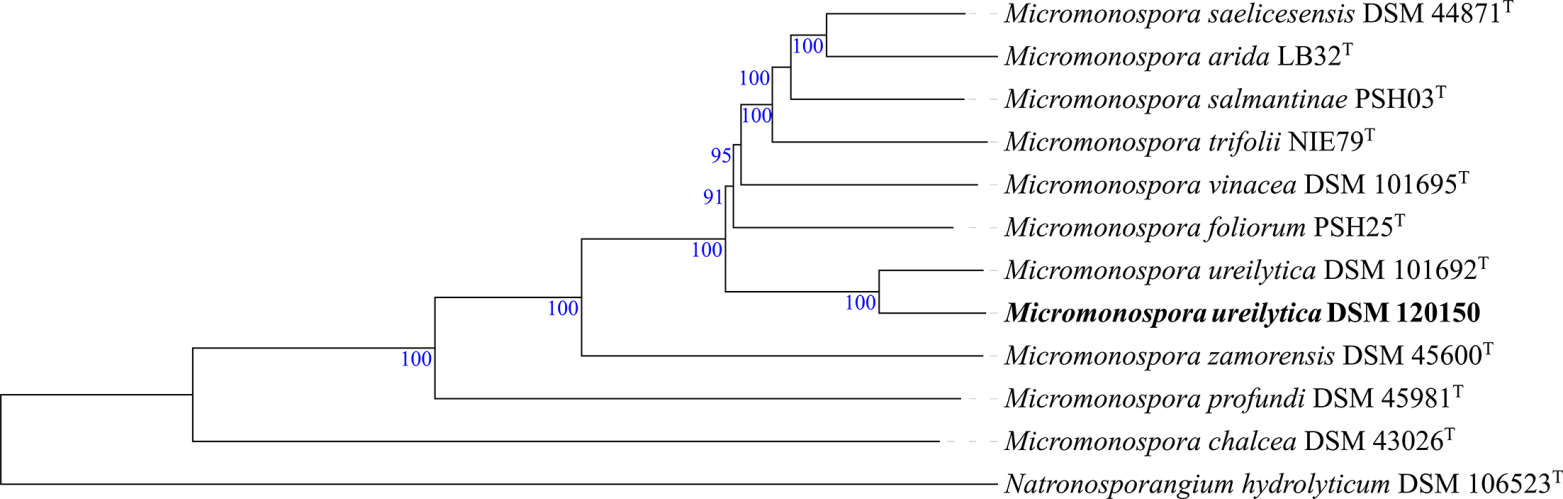
Phylogenetic tree of *M. ureilytica* DSM 120150 inferred with FastME 2.1.6.1 [41] from GBDP distances calculated from genome sequences. The branch lengths are scaled in terms of the GBDP distance formula *d*_5_. The tree was rooted at the midpoint [42] and visually edited using Interactive Tree of Life (iTOL) v.7.2.1 [43].

**Fig. 3.**
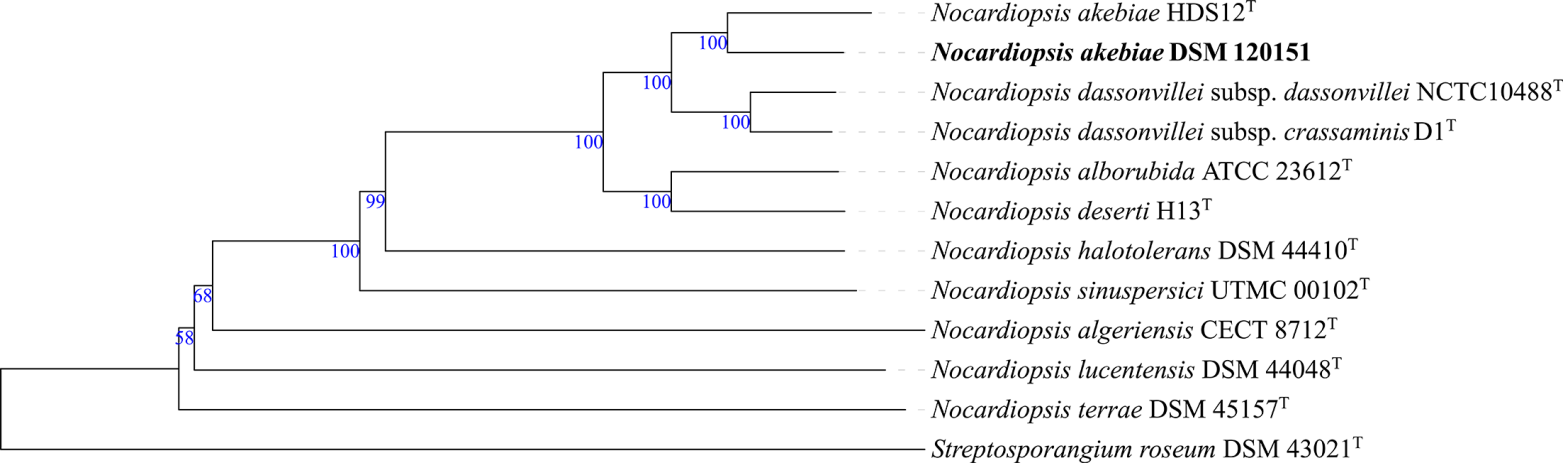
Phylogenetic tree of *N. akebiae* DSM 120151 inferred with FastME 2.1.6.1 [41] from GBDP distances calculated from genome sequences. The branch lengths are scaled in terms of the GBDP distance formula *d*_5_. The tree was rooted at the midpoint [42] and visually edited using Interactive Tree of Life (iTOL) v.7.2.1 [43].

Phylogenomic analysis based on whole-genome sequences and digital DNA–DNA hybridization (dDDH) (formula *d_4_*) was carried out with the Type Strain Genome Server [26,27]. The phylogenomic tree constructed from the *Streptomyces* genome sequences positioned strain *S. hypolithicus* DSM 41950^T^ in close association with *Streptomyces altiplanensis* HST21^T^, *Streptomyces flavidovirens* DSM 40150^T^ and *Streptomyces chryseus* JCM 4737^T^ [28,30], forming a well-supported subcluster, with dDDH values ranging from 29.9 to 31.1%. This study reports, for the first time, the genome sequence of *S. hypolithicus* DSM 41950^T^, designated as the type strain of *S. hypolithicus* in the original taxonomic description by Le Roes-Hill et al. [24] [24]. The strain *S. albidoflavus* DSM 120149 formed a distinct branch, closely related to a subcluster that included the type strain *S. albidoflavus* DSM 40455ᵀ [31], with a dDDH value of 64.7%. This subcluster also encompassed *Streptomyces limosus* NBRC 12790^T^, *Streptomyces sampsonii* NBRC 13083^T^ and *Streptomyces coelicolor* DSM 40233^T^ [31,33], with dDDH values ranging from 64.3 to 64.6%. All these strains are heterotypic synonyms of *S. albidoflavus*. To further assess the taxonomic position of DSM 120149, the average nucleotide identity (ANI) was analysed using the EzBioCloud tool ANI Calculator [34]. Strain DSM 120149 shared an ANI value of 95.97% with DSM 40455^T^, which is at the threshold for prokaryotic species delineation (95–96%) [35]. Strain *S. fildesensis* DSM 41987^T^ formed a distinct branch, closely related to a well-supported subclade comprising *Streptomyces soli* LAM7114^T^ and *Streptomyces oryziradicis* NEAU-C40^T^ [36,37], with dDDH values of 23.4% and 23.0%, respectively (Fig. 1). The strain DSM 41987^T^ was originally designated as the type strain of *S. fildesensis* by Li et al. [23] [23]. All dDDH values reported in this study were below the 70% species demarcation threshold, consistent with their assignment as distinct species. The Antarctic *Micromonospora* strain DSM 120150 formed a distinct branch closely related to *M. ureilytica* DSM 101692^T^ [38] (Fig. 2), with a dDDH value of 77.3%. In the *Nocardiopsis* phylogenomic tree, strain *N. akebiae* DSM 120151 formed a well-supported subbranch closely related to *N. akebiae* HDS12^T^ [39] (Fig. 3), with a dDDH value of 69.6% and an ANI value of 96.44%. The dDDH and ANI values between strains DSM 120150 and DSM 120151 and their closest relatives support their assignment to *M. ureilytica* and *N. akebiae*, respectively, although the borderline dDDH value observed for strain DSM 120151 suggests that additional genomic and phenotypic analyses would be required to further resolve its taxonomic status.

**Fig. 4.**
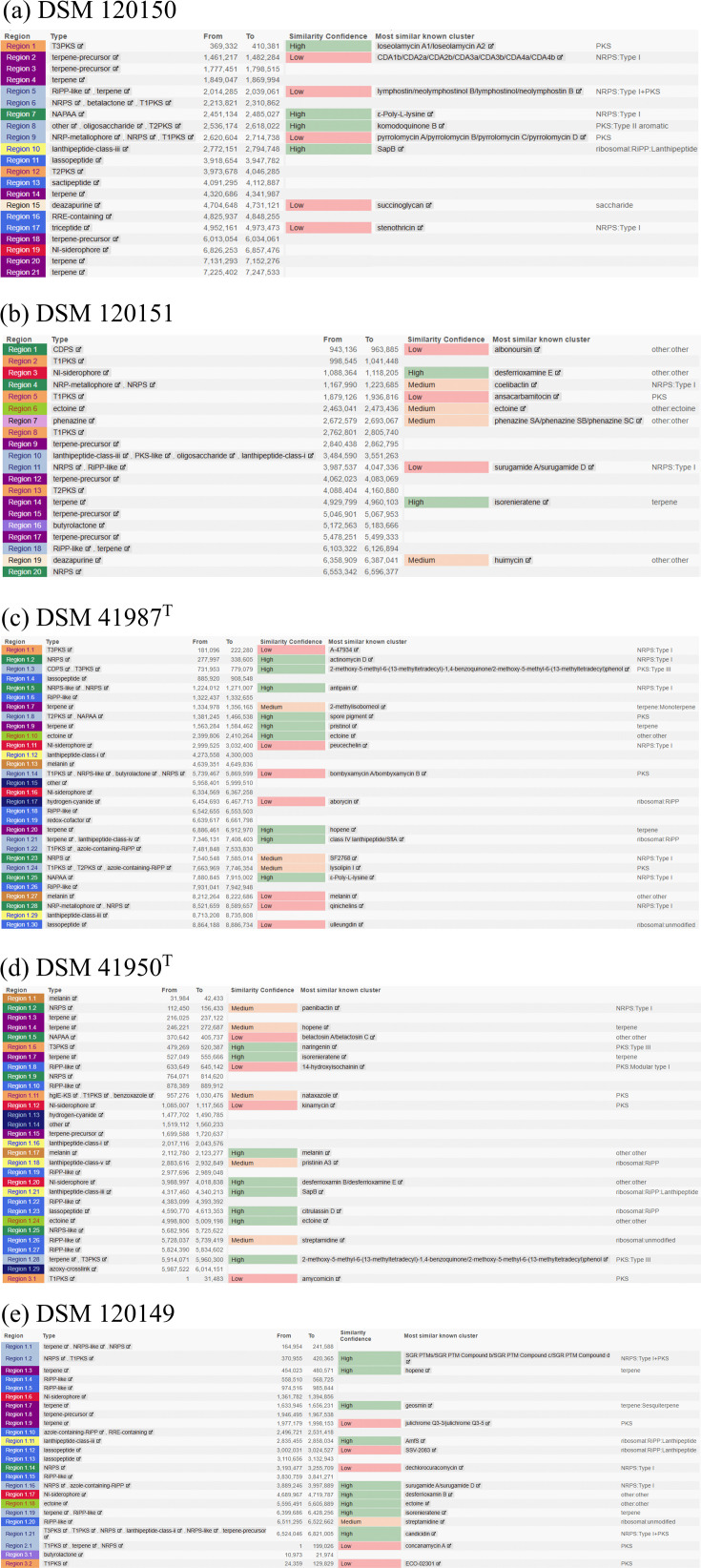
AntiSMASH output from Antarctic actinomycetes strains (**a**) *M. ureilytica* DSM 120150, (**b**) *N. akebiae* DSM 120151, (**c**) *S. fildesensis* DSM 41987^T^, (**d**) *S. hypolithicus* DSM 41950^T^ and (**e**) *S. albidoflavus* DSM 120149. Colour code according to antiSMASH v.8.0 [40].

To investigate the genetic potential for secondary metabolite production, the genome sequences of the five Antarctic strains were analysed with the bioinformatic web tool antiSMASH v.8.0 [40] for the abundance of biosynthetic gene clusters (BGCs). A total of 21, 20, 30, 30 and 24 BGCs were identified for strains DSM 120150, DSM 120151, DSM 41987^T^, DSM 41950^T^ and DSM 120149, respectively (Fig. 4a–e). The number and diversity of identified BGCs indicate a broad genetic repertoire associated with secondary metabolism.

This study provides high-quality genome sequence data for the five Antarctic actinomycete strains *M. ureilytica* DSM 120150, *N. akebiae* DSM 120151, *S. fildesensis* DSM 41987^T^, *S. hypolithicus* DSM 41950^T^ and *S. albidoflavus* DSM 120149 and their BGC cluster content associated with secondary metabolism.

## Abbreviations

ANI: average nucleotide identity
BGCs: biosynthetic gene clusters
dDDH: digital DNA–DNA hybridization
GBDP: Genome BLAST Distance Phylogeny

## References

[1] Avila C (2016). Biological and chemical diversity in Antarctica: from new species to new natural products. *Biodiversity*.

[2] Sivalingam P, Hong K, Pote J, Prabakar K (2019). Extreme environment *Streptomyces*: potential sources for new antibacterial and anticancer drug leads?. Int J Microbiol.

[3] Liao L, Su S, Zhao B, Fan C, Zhang J (2019). Biosynthetic potential of a novel antarctic actinobacterium *Marisediminicola antarctica* ZS314^T^ revealed by genomic data mining and pigment characterization. Mar Drugs.

[4] Núñez-Pons L, Avila C, Romano G, Verde C, Giordano D (2018). UV-Protective Compounds in Marine Organisms from the Southern Ocean. Mar Drugs.

[5] Li A-Z, Han X-B, Zhang M-X, Zhou Y, Chen M (2019). Culture-dependent and -independent analyses reveal the diversity, structure, and assembly mechanism of benthic bacterial community in the Ross Sea, Antarctica. Front Microbiol.

[6] Mast Y, Stegmann E (2019). Actinomycetes: the antibiotics producers. Antibiotics.

[7] Subramani R, Sipkema D (2019). Marine rare actinomycetes: a promising source of structurally diverse and unique novel natural products. Mar Drugs.

[8] Nouioui I, Boldt J, Zimmermann A, Makitrynskyy R, Pötter G (2024). Biotechnological and pharmaceutical potential of twenty-eight novel type strains of *Actinomycetes* from different environments worldwide. *Curr Res Microb Sci*.

[9] Quinn GA, Dyson PJ (2024). Going to extremes: progress in exploring new environments for novel antibiotics. *NPJ Antimicrob Resist*.

[10] Medeiros W, Kralova S, Oliveira V, Ziemert N, Sehnal L (2025). Antarctic bacterial natural products: from genomic insights to drug discovery. Nat Prod Rep.

[11] Handayani I, Saad H, Ratnakomala S, Lisdiyanti P, Kusharyoto W (2021). Mining Indonesian microbial biodiversity for novel natural compounds by a combined genome mining and molecular networking approach. Mar Drugs.

[12] Kieser T, Bibb MJ, Buttner MJ, Chater KF, Hopwood DA (2000). Practical Streptomyces Genetics.

[13] Mahmoud FM, Pritsch K, Siani R, Benning S, Radl V (2024). Comparative genomic analysis of strain *Priestia megaterium* B1 reveals conserved potential for adaptation to endophytism and plant growth promotion. Microbiol Spectr.

[14] Danecek P, Bonfield JK, Liddle J, Marshall J, Ohan V (2021). Twelve years of SAMtools and BCFtools. Gigascience.

[15] Fukasawa Y, Ermini L, Wang H, Carty K, Cheung MS (2020). LongQC: a quality control tool for third generation sequencing long read data. G3.

[16] van der Graaf-van Bloois L, Wagenaar JA, Zomer AL (2021). RFPlasmid: predicting plasmid sequences from short-read assembly data using machine learning. Microb Genom.

[17] Gurevich A, Saveliev V, Vyahhi N, Tesler G (2013). QUAST: quality assessment tool for genome assemblies. Bioinformatics.

[18] Li W, O’Neill KR, Haft DH, DiCuccio M, Chetvernin V (2021). RefSeq: expanding the prokaryotic genome annotation pipeline reach with protein family model curation. Nucleic Acids Res.

[19] Parks DH, Imelfort M, Skennerton CT, Hugenholtz P, Tyson GW (2015). CheckM: assessing the quality of microbial genomes recovered from isolates, single cells, and metagenomes. Genome Res.

[20] Chalita M, Kim YO, Park S, Oh H-S, Cho JH (2024). EzBioCloud: a genome-driven database and platform for microbiome identification and discovery. Int J Syst Evol Microbiol.

[21] Carro L, Castro JF, Razmilic V, Nouioui I, Pan C (2019). Uncovering the potential of novel micromonosporae isolated from an extreme hyper-arid Atacama Desert soil. Sci Rep.

[22] Camacho Pozo MI, Wieme AD, Pérez SR, Llauradó Maury G, Snauwaert C (2020). *Nocardiopsis dassonvillei* subsp. *crassaminis* subsp. nov., isolated from freshwater sediment, and reappraisal of *Nocardiopsis alborubida* Grund and Kroppenstedt 1990 emend. Nouioui *et al*. 2018. Int J Syst Evol Microbiol.

[23] Li J, Tian XP, Zhu TJ, Yang LL, Li WJ (2011). *Streptomyces fildesensis* sp. nov., a novel streptomycete isolated from Antarctic soil. Antonie Van Leeuwenhoek.

[24] Le Roes-Hill M, Rohland J, Meyers PR, Cowan DA, Burton SG (2009). *Streptomyces hypolithicus* sp. nov., isolated from an Antarctic hypolith community. Int J Syst Evol Microbiol.

[25] Somphong A, Polyiam W, Suriyachadkun C, Sripreechasak P, Harunari E (2024). *Streptomyces pyxinae* sp. nov. and *Streptomyces pyxinicus* sp. nov. isolated from lichen *Pyxine cocoes* (Sw.) Nyl. Int J Syst Evol Microbiol.

[26] Meier-Kolthoff JP, Göker M (2019). TYGS is an automated high-throughput platform for state-of-the-art genome-based taxonomy. Nat Commun.

[27] Meier-Kolthoff JP, Carbasse JS, Peinado-Olarte RL, Göker M (2022). TYGS and LPSN: a database tandem for fast and reliable genome-based classification and nomenclature of prokaryotes. Nucleic Acids Res.

[28] Cortés-Albayay C, Dorador C, Schumann P, Schniete JK, Herron P (2019). *Streptomyces altiplanensis* sp. nov., an alkalitolerant species isolated from Chilean Altiplano soil, and emended description of *Streptomyces chryseus* (Krasil’nikov *et al*. 1965) Pridham 1970. Int J Syst Evol Microbiol.

[29] Pridham TG, Hesseltine CW, Benedict RG (1958). A guide for the classification of streptomycetes according to selected groups; Placement of strains in morphological sections. Appl Microbiol.

[30] Pridham GT (1970). New names and new combinations in the order Actinomycetales Buchanan 1917. *Technical Bulletins*.

[31] Waksman S, Henrici A, Breed R, Murray E, Hitchens A (1948). Bergey’s Manual of Determinative Bacteriology. 6th ed.

[32] Lindenbein W (1952). Über einige chemisch interessante Aktinomycetenstämme und ihre Klassifizierung. Archiv Mikrobiol.

[33] Waksman S, Lechevalier H (1953). Guide to the Classification and Identification of the Actinomycetes and Their Antibiotics.

[34] Yoon S-H, Ha S-M, Lim J, Kwon S, Chun J (2017). A large-scale evaluation of algorithms to calculate average nucleotide identity. Antonie Van Leeuwenhoek.

[35] Richter M, Rosselló-Móra R (2009). Shifting the genomic gold standard for the prokaryotic species definition. Proc Natl Acad Sci U S A.

[36] Xing J, Jiang X, Kong D, Zhou Y, Li M (2020). *Streptomyces soli* sp. nov., isolated from birch forest soil. Arch Microbiol.

[37] Li C, Cao P, Jiang M, Sun T, Shen Y (2020). *Streptomyces oryziradicis* sp. nov., a novel actinomycete isolated from rhizosphere soil of rice (*Oryza sativa* L.). Int J Syst Evol Microbiol.

[38] Carro L, Riesco R, Spröer C, Trujillo ME (2016). *Micromonospora ureilytica* sp. nov., *Micromonospora noduli* sp. nov. and *Micromonospora vinacea* sp. nov., isolated from *Pisum sativum* nodules. Int J Syst Evol Microbiol.

[39] Mo P, Zhou J, Zhou F, He J, Zou W (2022). *Nocardiopsis akebiae* sp. nov., a novel endophytic actinomycete isolated from fruits of *Akebia trifoliata*. Arch Microbiol.

[40] Blin K, Shaw S, Vader L, Szenei J, Reitz ZL (2025). antiSMASH 8.0: extended gene cluster detection capabilities and analyses of chemistry, enzymology, and regulation. Nucleic Acids Res.

[41] Lefort V, Desper R, Gascuel O (2015). FastME 2.0: a comprehensive, accurate, and fast distance-based phylogeny inference program. Mol Biol Evol.

[42] Farris JS (1972). Estimating phylogenetic trees from distance matrices. Am Nat.

[43] Letunic I, Bork P (2024). Interactive Tree of Life (iTOL) v6: recent updates to the phylogenetic tree display and annotation tool. Nucleic Acids Res.

